# Analyzing COVID‐19 Using Multisource Data: An Integrated Approach of Visualization, Spatial Regression, and Machine Learning

**DOI:** 10.1029/2021GH000439

**Published:** 2021-08-01

**Authors:** Chao Wu, Mengjie Zhou, Pengyu Liu, Mengjie Yang

**Affiliations:** ^1^ School of Geographic and Biologic Information Nanjing University of Posts and Telecommunications Nanjing China; ^2^ Smart Health Big Data Analysis and Location Services Engineering Lab of Jiangsu Province Nanjing University of Posts and Telecommunications Nanjing China; ^3^ College of Resources and Environmental Science Hunan Normal University Changsha China; ^4^ Key Laboratory of Geospatial Big Data Mining and Application Changsha China

**Keywords:** COVID‐19, spatial‐temporal patterns, visualization, mixed GWR, XGBoost, geographical perspective

## Abstract

Coronavirus disease 2019 (COVID‐19), caused by severe acute respiratory syndrome coronavirus 2, was first identified in Wuhan, China, in December 2019. As the number of COVID‐19 infections and deaths worldwide continues to increase rapidly, the prevention and control of COVID‐19 remains urgent. This article aims to analyze COVID‐19 from a geographical perspective, and this information can provide useful insights for rapid visualization of spatial‐temporal epidemic information and identification of the factors important to the spread of COVID‐19. A new type of vitalization method, called the point grid map, is integrated with calendar‐based visualization to show the spatial‐temporal variations in COVID‐19. The combination of mixed geographically weighted regression (mixed GWR) and extreme gradient boosting (XGBoost) is used to identify the potential factors and the corresponding importance. The visualization results clearly reflect the spatial‐temporal patterns of COVID‐19. The quantified results reveal that the impact of population outflow from Wuhan is the most important factor and indicate statistically significant spatial heterogeneity. Our results provide insights into how multisource big geodata can be employed within the framework of integrating visualization and analytical methods to characterize COVID‐19 trends. In addition, this work can help understand the influential factors for controlling and preventing epidemics, which is important for policy design and effective decision‐making for controlling COVID‐19. The results reveal that one of the most effective ways to control COVID‐19 include controlling the source of infection, cutting off the transmission route, and protecting vulnerable groups.

## Key Points

- A point grid map is integrated with calendar‐based visualization to represent the spatial‐temporal variations of COVID‐2019 in China
- Mixed geographically weighted regression and extreme gradient boost are employed to identify the factors important to the spread of COVID‐19
- The framework integrating visualization, spatial regression, and machine learning can be applied to spatial epidemiology studies elsewhere

## 1 Introduction

Recently, coronavirus disease 2019 (COVID‐19) suddenly broke out in Wuhan, Hubei Province, China (Chen et al., 2020; Huang et al., 2020; Sohrabi et al., 2020). As of April 30, 2020, the total number of confirmed cases in mainland China reached 82,858, of whom 4,633 died and 77,578 recovered according to the report released by the Chinese Center for Disease Control and Prevention. The grim situation of COVID‐19 had a comprehensive and profound impact on people's daily lives, economic development, and social harmony. Faced with the ferocious COVID‐19 epidemic, the Chinese government has quickly and decisively taken a series of effective measures, including the construction of makeshift hospitals, the closure of cities, the isolation of patients, the initiation of a primary response and community‐based management, and has achieved remarkable results. Owing to the above effective and rapid measures taken by the Chinese government, the COVID‐19 outbreak in China has been effectively controlled, and many cities have resumed work and production.

Notably, the closure of Wuhan on January 23 is one of the most powerful measures against the spread of COVID‐19. Studies have shown that the lockdown has effectively limited the growth and spread of COVID‐19 in China (Kraemer et al., 2020; Tian et al., 2020). However, the migration of people from Wuhan before the 2020 Spring Festival holiday, especially before banned travel to and from Wuhan, inevitably led to an increasing number of infections across the country (Wu, Leung, & Leung, 2020). Moreover, Li et al. (2020) indicate that in the early stage of the epidemic, the time from infection to onset is approximately 5.2 days, which allows infected patients continue to have contact with people, including relatives, friends, and even passers‐by, without any awareness. Therefore, migration and contact within cities can also lead to the transmission of COVID‐19. Although China has basically controlled the COVID‐19 epidemic, China still cannot relax vigilance in the context of the COVID‐19 outbreak worldwide. Different from the perspective of biopharmaceutical research, analysis of the spatiotemporal patterns and influential factors of COVID‐19 from a geographical perspective can effectively help to curb the spread of the disease, quantify the risk factors for COVID‐19 and improve resource allocation and emergency decision‐making.

Analyzing the spatial‐temporal pattern of COVID‐19 is of great significance for the treatment, prevention, and control of COVID‐19. Visualization has played an important role in epidemiology research and outbreak surveillance (Preim & Lawonn, 2020), and it offers promising methods to support the spatial‐temporal analysis of COVID‐19. In existing studies, different visualization techniques (e.g., histograms, cartograms, choropleth maps, dot maps, and time series plots) combined with analytics components (e.g., hierarchical clustering and multidimensional scaling) have been used to observe the reported COVID‐19 confirmed, death, and recovered cases (Dey et al., 2020); understand the spread and expansion of COVID‐19 (P. Gao et al., 2020); compare the evolution of COVID‐19 in different countries (Machado & Lopes, 2020); and monitor people's reaction to social distancing policies (S. Gao et al., 2020). However, there is a lack of visualization and analysis for both the overview and detailed spatial‐temporal patterns of COVID‐19.

In addition, exploring the influential factors of COVID‐19 is equally as important in providing policymakers useful insights for targeted COVID‐19 interventions. A number of researchers have explored the factors affecting the COVID‐19 epidemic from the aspects of population mobility (Kraemer et al., 2020; Tian et al., 2020), air quality (Ogen, 2020; Zhu et al., 2020), and smoking (Cai, 2020; Vardavas & Nikitara, 2020), as well as the atmospheric (Ma et al., 2020; Oliveiros et al., 2020; Qi et al., 2020) and the socioeconomic environments (Mollalo et al., 2020). Based on the reported confirmed cases, the majority of the locally infected patients outside Wuhan had a Wuhan travel history. Therefore, human mobility can be regarded as one of the main factors of COVID‐19 spread. On the one hand, population flow can effectively reflect the functional connection between cities, and the function of each city is also different, which indicates that human mobility can have spatially different effects on the spread of COVID‐19 spread. On the other hand, the movement of people within cities after closure will also lead to the spread of COVID‐19 and the conditions of cities' own responses to the outbreak are different, such as the proportion of the susceptible population and medical resources. However, the spatial influence mechanisms of human mobility, including influential strength and effects, on the spread of COVID‐19 over China remain unclear.

Above all, this article aims to analyze the COVID‐19 epidemic at the city‐level in China from a geographical perspective. This article integrates an analysis framework including a point grid map embedded with the calendar view, mixed geographically weighted regression (mixed GWR), and extreme gradient boosting (XGBoost) to study the spatial and temporal characteristics of COVID‐19 using multisource geodata. The proposed framework integrating visualization method, spatial regression model, and machine learning technology is generalized, which can be extended to the study of spatial epidemiology in other regions. Our proposed framework can provide a geographical insight to understand spatial epidemics, which can provide additional advice on the study of epidemiology from pathology. Our work contributes to the existing literature in the following aspects. Specifically, (a) we combined a new map format, namely, the point grid map (Zhou et al., 2016, 2017), with calendar‐based visualization to visualize the daily confirmed cases of COVID‐19 in each city, which can effectively and intuitively reflect the spatial and temporal dynamics and evolution of COVID‐19. (b) Mixed GWR is used to explore the spatial effects of influential factors on COVID‐19 while considering the significance of the spatial heterogeneity of the influential factors. On the one hand, the heterogeneity test can truly reflect real change characteristics of COVID‐19 and related influential factors, which can reduce the risk of overfitting. On the other hand, the mixed GWR can effectively measure the differences in the influence degree of the same factor in different spatial locations. (c) XGBoost is used to reveal the nonlinear relationships between COVID‐19 and its influential factors, which can identify the importance of influential factors. The mixed GWR and XGBoost, which are two complementary methods balancing the linear and nonlinear relationships, are integrated to comprehensively identify the relationships between the influential factors and COVID‐19 at a city‐level in China. The results of this article can provide new insights to dynamically monitor COVID‐19 and facilitate better public participation to fight the epidemic. Moreover, this article can also provide useful suggestions and advice to control and alleviate the COVID‐19 epidemic in other countries and regions.

## 2 Materials and Methods

### 2.1 Data and Variables Definition

This article collected COVID‐19 cases from Harvard Dataverse (Hu et al., 2020). The data can be freely downloaded from their website (https://doi.org/10.7910/DVN/MR5IJN). In the temporal dimension, the data of COVID‐19 cases are currently updated daily, and this article calculated the cumulative number of confirmed cases of COVID‐19 from January 15 to April 30, 2020. The confirmed cases linked to the city where test was conducted. In the spatial dimension, the daily confirmed cases of COVID‐19 are counted and aggregated at the city‐level in China (prefectural‐level cities). A prefectural‐level city is an administrative division of China, ranking below a province and above a county in China's administrative structure. However, due to the lack of data, several cities (or regions) are not represented, including Macao, Taiwan, Sansha, Baisha Li Autonomous County, Tunchang, Wuzhishan, Tumushuke, Tiemenguan, Wujiaqu, Shuanghe, Beitun, Shihezi, Alaer, Kunyu, Kekedala, and Chongzuo. In this article, we aim to explore the relationships between the cumulative confirmed cases of COVID‐19 and the influential factors.

Most of the early confirmed cases outside Wuhan had a history of travel to Wuhan from December 2019 to January 2020. Beyond that, some local confirmed cases are infected from activities within the city. In view of the foregoing, we selected three variables as the influential factors of COVID‐19 transmission. Based on the time of Wuhan's closure (i.e., January 23, 2020), the population mobility index from Wuhan before closure is taken as an influential factor called the Wuhan migration index. The mobility data from Wuhan were derived from Baidu Qianxi (https://qianxi.baidu.com/2020/). The migration index from Wuhan to other cities used in this paper can reflect the population scale of moving out of Wuhan, and the cities can be compared horizontally. The mobility data used in the mixed GWR and XGBoost models are the average migration index values before the closure of Wuhan on January 23. Baidu Qianxi also provides data describing the intensity of urban internal travel, which is the indexation result of the ratio of the number of people with travel behavior to the total number of residents in a city. Then, the mobility population is obtained by multiplying the intensity of urban internal travel by the total population. This article uses the average intracity mobility population after the closure of Wuhan as the proxy of urban vibrancy, called intracity vibrancy. Finally, populations susceptible to COVID‐19 in cities are measured using the proportion of the elderly population at the province level for data availability (Wu & McGoogan, 2020), and this information is obtained from the 2019 China Statistical Year Book.

### 2.2 Visualization Methods

Visualization is a powerful tool to help humans discover the hidden patterns of spatial‐temporal data visually and intuitively (Robinson et al., 2017). We combined a point grid map with calendar‐based visualization (Van Wijk & Van Selow, 1999; Zhou et al., 2016, 2017) to visualize the spatial‐temporal patterns of confirmed COVID‐19 cases and their potential factors at the city‐level.

#### 2.2.1 Calendar‐Based Visualization

**Figure 1:**
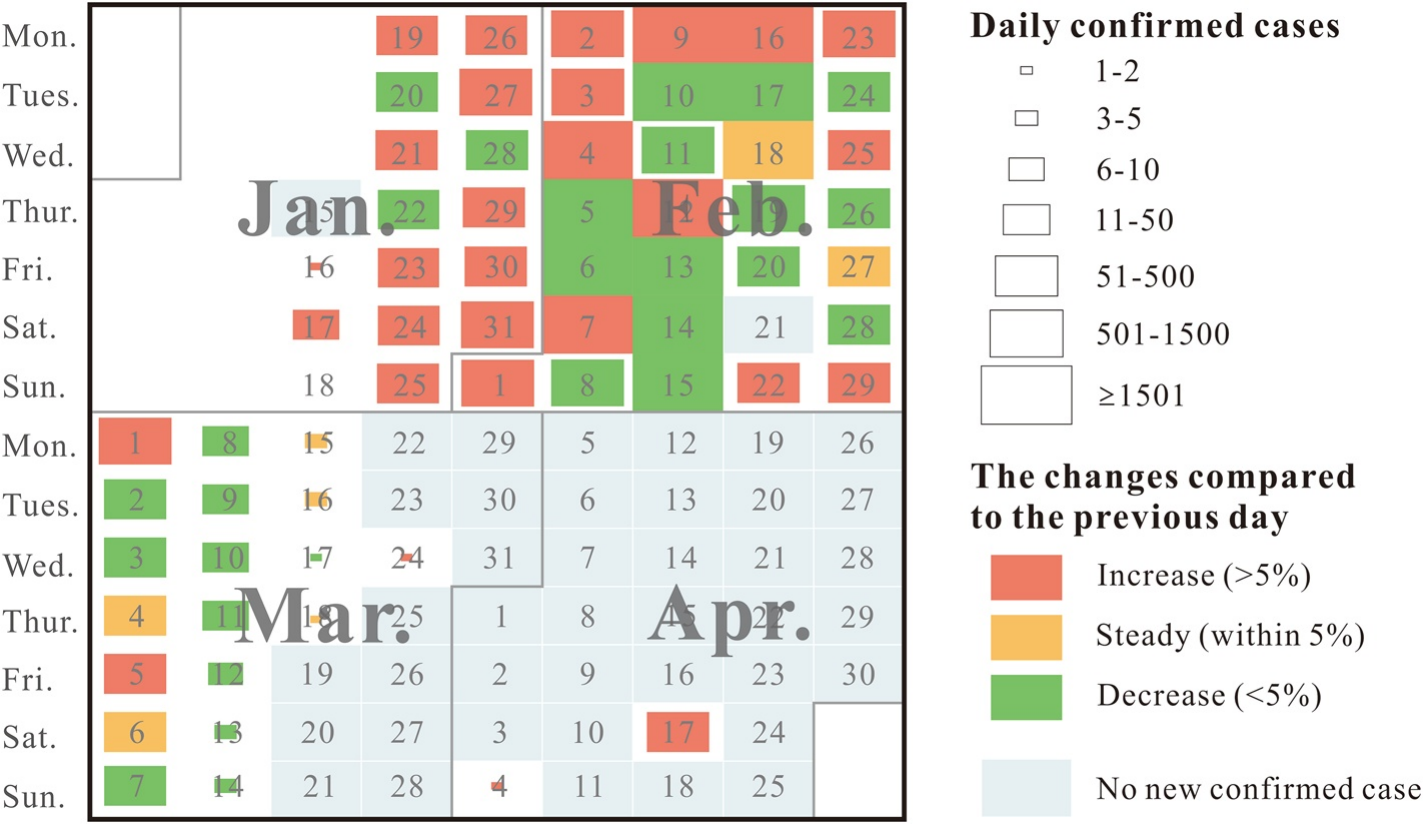
Calendar‐based visualization of confirmed cases from January 15 to April 30 in Wuhan.

Calendar‐based visualization, which represents daily data on a calendar, is an effective visualization method to represent time series data (Van Wijk & Van Selow, 1999). Days in the calendar can be colored or placed with symbols corresponding to the daily information. We use calendar‐based visualization to visualize daily confirmed cases in each city. Temporal trends and patterns of daily confirmed cases can be easily identified through calendar‐based visualization. Figure 1 illustrates the results of calendar‐based visualization of confirmed cases in Wuhan. Symbols (rectangles) are placed in the calendar to represent daily confirmed cases. The sizes of the rectangles in days represent the number of confirmed cases. The color of rectangles represents the changes in the number of confirmed cases compared to the previous day. Here, red indicates an increase by greater than 5%, yellow indicates a change within 5%, and green indicates a decrease by greater than 5%. The distribution of daily confirmed cases is extremely skewed. The geometrical interval classification method is useful for visualizing data that are not distributed normally or when the distribution is extremely skewed. We used the geometrical interval classification method to classify daily confirmed cases and then modified the break value to ensure that the break value was close to a round number, such as 5, 10, 50, 500, and 1,500. A sudden spike was noted on April 17. The sudden spike was caused by a revision of the number of confirmed COVID‐19 cases in Wuhan. Indeed, no new confirmed cases were reported in Wuhan on April 17.

#### 2.2.2 Point Grid Map

**Figure 2:**
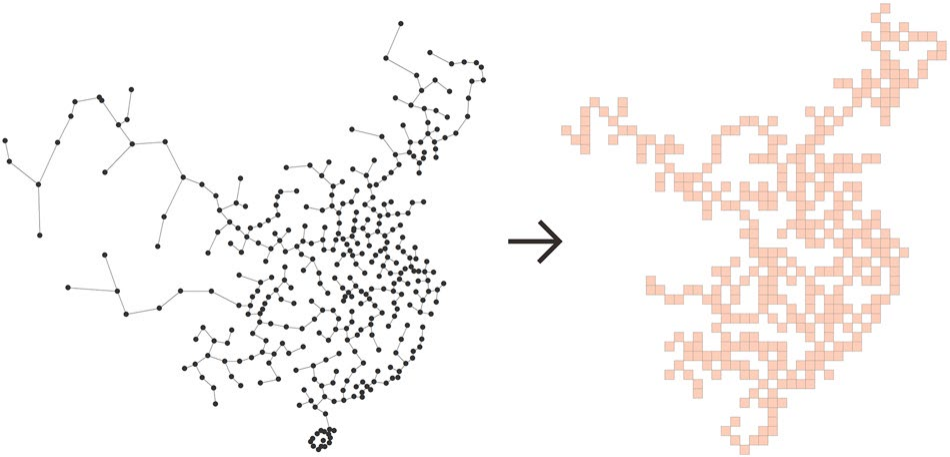
Point grid map representing cities in China.

A point grid map transforms input cities into a grid in which each grid cell represents a city while maintaining the relative approximate position of each city (Zhou et al., 2017). Two steps are employed to construct a point grid map of the cities in China: (a) extract the structure of cities and (b) transform the cities into a grid based on the structure, as shown in Figure 2. Due to the lack of data, Hong Kong, Macao, Taiwan, Sansha, Baisha Li Autonomous County, Tunchang, Wuzhishan, Tumushuke, Tiemenguan, Wujiaqu, Shuanghe, Beitun, Shihezi, Alaer, Kunyu, Kekedala, and Chongzuo are not represented in the point grid map. Data associated with the cities can be displayed on the point grid map with different colors, symbols, or diagrams. Point grid map has several advantages. First, the point grid map shows a clear appearance and can avoid the overlaps of symbols or diagrams. Second, comparisons between different cities and between different data represented by a point grid map are very easy, as grid cells are aligned in a point grid map.

### 2.3 Mixed Geographically Weighted Regression

As one of the effective extension models of ordinary least squares (OLS) regression, GWR offers the greatest advantage in solving the problem of spatial heterogeneity (Brunsdon et al., 1996; Fotheringham et al., 2003; Wu et al., 2019; Wu, Peng, Ma, Li, & Rao, 2020). However, in practical applications, it is necessary to determine whether the spatial heterogeneity detected by GWR is caused by the change in spatial relationships truly contained in the original data, random factors, or incorrect model settings (Fotheringham et al., 2003). The statistical test of spatial heterogeneity is of great significance to verify the rationality and correctness of the selected model and prevent the phenomenon of overfitting (Mei et al., 2004, 2006). Based on the results of statistical analysis, the influence of some explanatory variables on response variables may be global, but there are also some explanatory variables that change locally. In view of the above, compared with GWR, mixed GWR can better reflect the true distribution of data, and prevent the result of overfitting, which can effectively reveal the real relationship between COVID‐19 and influential factors. Therefore, GWR is extended to mixed GWR, which allows the coexistence of spatially varying coefficients and global coefficients (Fotheringham et al., 2003; Mei et al., 2004; Wu et al., 2019; Wu, Leung, & Leung, 2020).

(1) $$y_{i}=\sum_{k}\;\beta_{k}\left(u_{i},v_{i}\right)x_{ik}+\sum_{l}\;\beta_{l}x_{il}+\varepsilon_{i},\;i=1,\;2\;\ldots ,\;n,$$

Here $\beta_{l}$ represent constant parameters that are global and fixed, and $\beta_{k}$ ($u_{i},v_{i}$) denotes the locally varying estimated coefficients. If k=0, Equation 1 is OLS; if l=0, Equation 1 is GWR. The essence of mixed GWR is a semiparametric spatial regression model with variable coefficients. Due to the existence of constant and local parameters, the locally weighted least squares method is no longer applicable to the calibration of mixed GWR. Therefore, two common methods were proposed to calibrate the mixed GWR, namely, the backfitting algorithm and two‐step estimation method, as noted in previous studies (Mei et al., 2004, 2006; Wu et al., 2019; Wu, Peng, Ma, Li, & Rao, 2020; Yu et al., 2020). For the calibration of GWR, the selection of the optimal bandwidth is important. The larger the spatial bandwidth, the larger the neighbor selected by regression point parameter estimation, and the lower the spatial heterogeneity. In this study, the minimum corrected Akaike information criterion (AICc) proposed by Fotheringham et al. (2003) is used to select the optimal bandwidth. The application of mixed GWR can reveal the real relationships between COVID‐19 and the influential factors, including global and local relationships based on the statistical test results of spatial heterogeneity.

### 2.4 XGBoost Regression Model

XGBoost is an extensible machine learning system gradient tree boosting that is widely applied to study issues related to regression, classification, rank, and prediction (Chen & Guestrin, 2016). One of the most salient advantages is that XGBoost is scalable in all scenarios, which depends on several important systems and algorithmic optimizations. XGBoost results can penalize the complexity of the model and help smooth final learned weights to prevent overfitting (Zhai & Chen, 2018). The iteratively optimized objective function of XGBoost is calculated as follows:

(2) $${\text{Obj}}^{\left(t\right)}=\sum_{i=1}^{n}\;l\left(y_{i}{\hat{y}}_{i}^{\left(t-1\right)}+f_{t}\left(x_{i}\right)\right)+\Omega \left(f_{t}\right)+\text{constant,}$$

Then, a second‐order Taylor approximation can be used to approximate the objective function as noted in Equation 2:

(3) $${\text{Obj}}^{\left(t\right)}≃\sum_{i=1}^{n}\left[l\left(y_{i}{\hat{y}}_{i}^{\left(t-1\right)}\right)+g_{i}f_{t}\left(x_{i}\right)+\frac{1}{2}h_{i}f_{t}^{2}\left(x_{i}\right)\right]+\Omega \left(f_{t}\right)+\text{constant},$$

where *l* is a differentiable convex loss function. $\Omega \left(f_{t}\right)$ is the regular item. $g_{i}=\partial_{{\hat{y}}_{i}^{\left(t-1\right)}}l\left(y_{i},{\hat{y}}_{i}^{\left(t-1\right)}\right)$, $h_{i}=\partial_{{\hat{y}}_{i}^{\left(t-1\right)}}^{2}l\left(y_{i},{\hat{y}}_{i}^{\left(t-1\right)}\right)$, and f(x) represent one of the regression trees. Equation 2 obtains the function using Taylor that expansion is essentially decoupled, and the result is that it increases the applicability of XGBoost to make it possible to use a loss function for regression on demand. XGBoost calculates which predictor is chosen as the split point based on the gain of the structure fraction, and the importance of a predictor is the sum of the times it appears in all the trees. In other words, the more a predictor is used to build a decision tree in the model, the more important the predictor is. Therefore, this article adopts XGBoost to reveal the nonlinear relationships between COVID‐19 and influential factors. The results can identify the order of importance of the influential factors of COVID‐19 in China, which supplements the results of mixed GWR and will improve the interpretability of the prediction model. In summary, these three methods, namely, a point grid map embedded with the calendar, mixed GWR, and XGBoost, are used to explore the trend and characteristics of COVID‐19 at the city level and identify the complex relationship between variables and COVID‐19 from different perspectives. By comparing the results, we can understand the relationship between variables and housing prices more comprehensively and thoroughly.

## 3 Spatial‐Temporal Visualizations of COVID‐19

**Figure 3:**
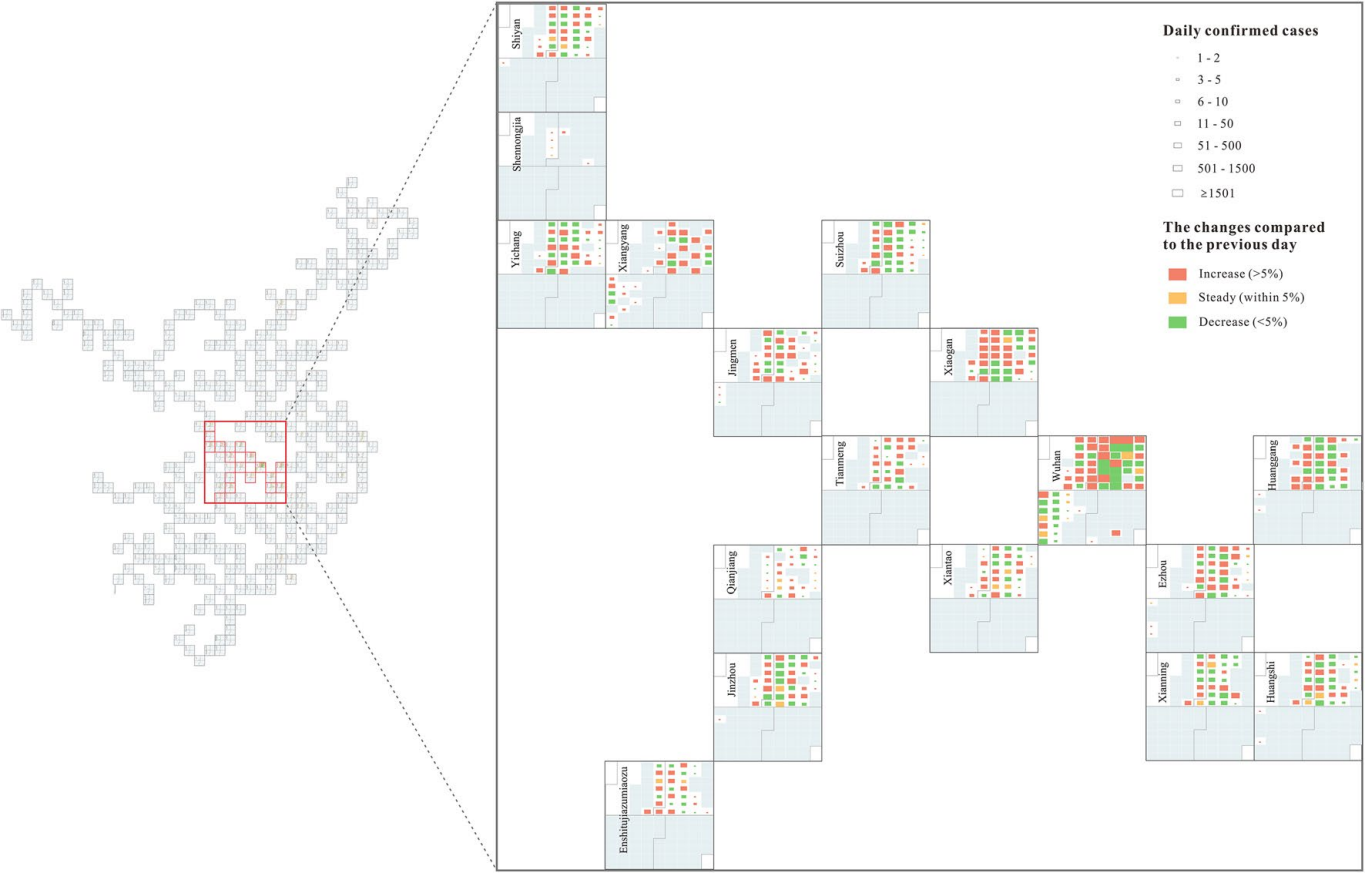
The spatial‐temporal visualization of coronavirus disease 2019, an enlarged view of Hubei Province.

This article uses calendar‐based visualization to represent daily confirmed cases of COVID‐19 in each city and uses a point grid map to arrange the cities based on relative approximate positions. The visualization result is shown in Figure S1. A tool based on the ArcGIS Engine was developed to construct a point grid map. ArcMap was used to visualize the daily confirmed cases. Finally, CorelDRAW was used for cartographic enhancement. We also made an online map (https://geocartolab.github.io/geocartolab/COVID.html) based on scalable vector graphics and JavaScript. In general, calendars representing confirmed cases of cities in western China are generally empty, which demonstrates that the number of people infected with COVID‐19 in the western region of China is relatively small and the overall situation is good. Symbols on the calendars of cities in Hubei Province, especially in Wuhan, are much larger and greater than those in other cities, revealing a serious COVID‐19 situation, as shown in Figure 3. In Wuhan, from late January to early March, especially in February, the number of daily confirmed cases was high. On February 4, there were 1,967 confirmed cases, and the number of confirmed cases peaked at 13,436 on February 12. From March 7 to 18, the color of rectangles representing daily confirmed cases is green or yellow, indicating a decrease or a steady of daily confirmed cases compared to the previous day. On March 17 and 18, there was only one daily confirmed case on each day. Then, there were no new confirmed cases for a relatively long time, revealing that the COVID‐19 outbreak has been effectively controlled. This level of control was dependent upon a range of Chinese government policies. In addition to Wuhan, Xiaogan, Huanggang, and Jingmen were also minimally affected by COVID‐19, especially in February. Other cities in Hubei Province show a similar temporal pattern. Specifically, daily confirmed cases are mainly concentrated from late January to February, and almost all of the grids are empty in March and April, indicating no new confirmed cases.

**Figure 4:**
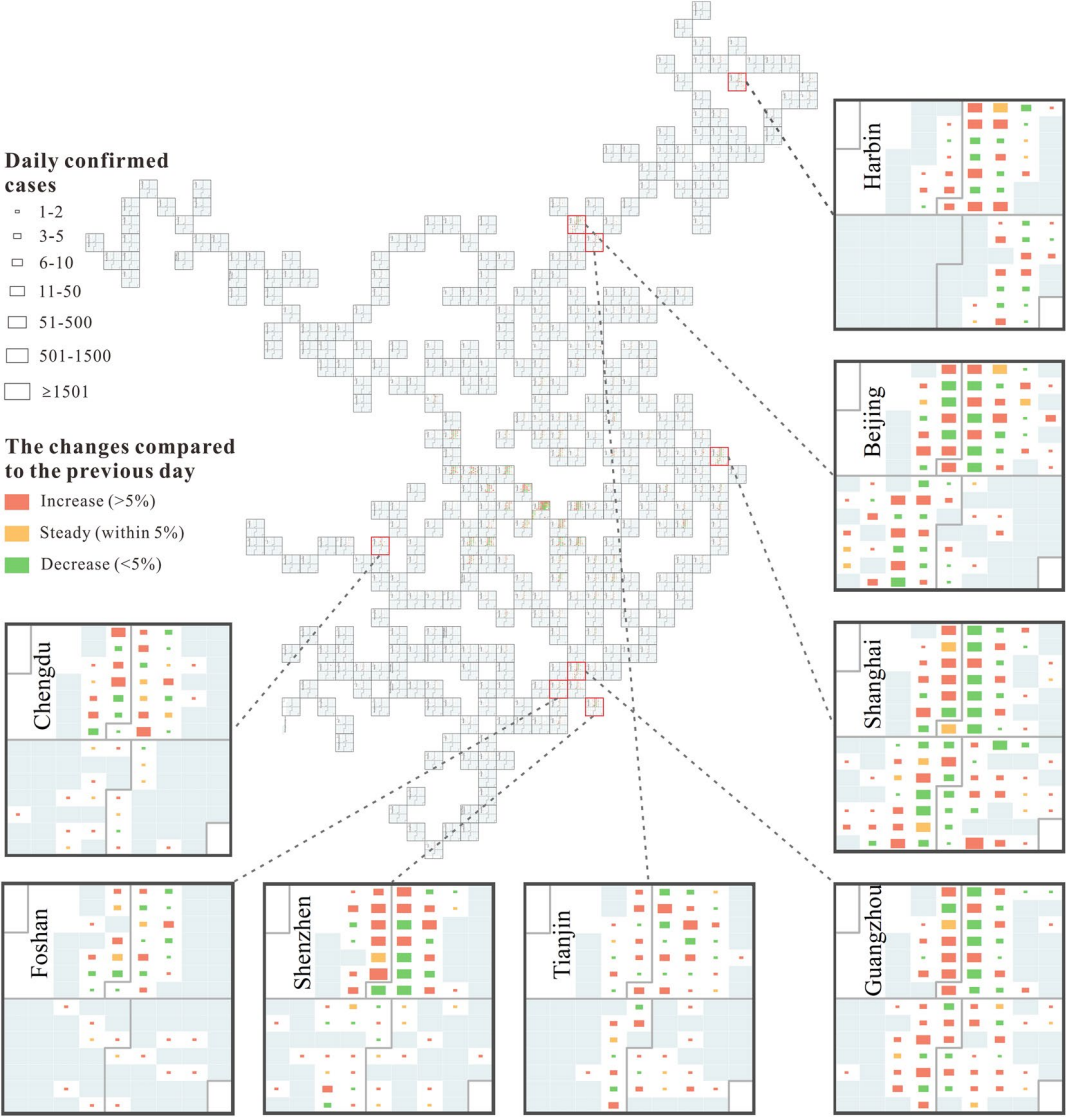
The spatial‐temporal visualization of coronavirus disease 2019, an enlarged view of some cities.

Outside Hubei Province, symbols representing daily confirmed cases were mainly distributed from late January to mid‐February in calendars for most of the cities, after which the calendars were empty. However, there were also some exceptions. For example, Beijing, Shanghai, Guangzhou, Harbin, Shenzhen, Foshan, Chengdu, and Tianjin showed different temporal patterns, as shown in Figure 4. China was the first country to experience and control the COVID‐19 pandemic, whereas China was later exposed to high imported case risk after the epidemic centers switched to Europe and then the US (Zhang et al., 2020). For cities with unusual spatiotemporal patterns, the confirmed cases are generally all imported cases and import associated cases in March and April. For Shenzhen, Foshan, Chengdu, and Tianjin, symbols in calendars are scattered in March and April, revealing that there are still confirmed cases in this period. For Harbin, zero domestic infections were reported for a relatively long period of consecutive days. However, since Harbin reported newly confirmed cases in early April, clusters of COVID‐19 infections have frequently been reported. The occurrence of COVID‐19 infection clusters was due to poorly implemented control measures by officials from Harbin. Beijing, Shanghai, Guangzhou, and Shenzhen are “first‐tier cities.” In particular, Beijing, Shanghai, and Guangzhou have superior international air connectivity compared with other cities in China. Thus, Beijing, Shanghai, and Guangzhou were facing the highest imported case risk. As shown in Figure 4, for Beijing, symbols representing daily confirmed cases were mainly distributed from late January to mid‐April in the calendar. For Shanghai, new confirmed cases occurred from late January to April. For Guangzhou, there were few confirmed cases from late February to mid‐March; however, the symbols in the calendar became larger from mid‐March, indicating that the situation of new confirmed cases worsened slightly.

**Figure 5:**
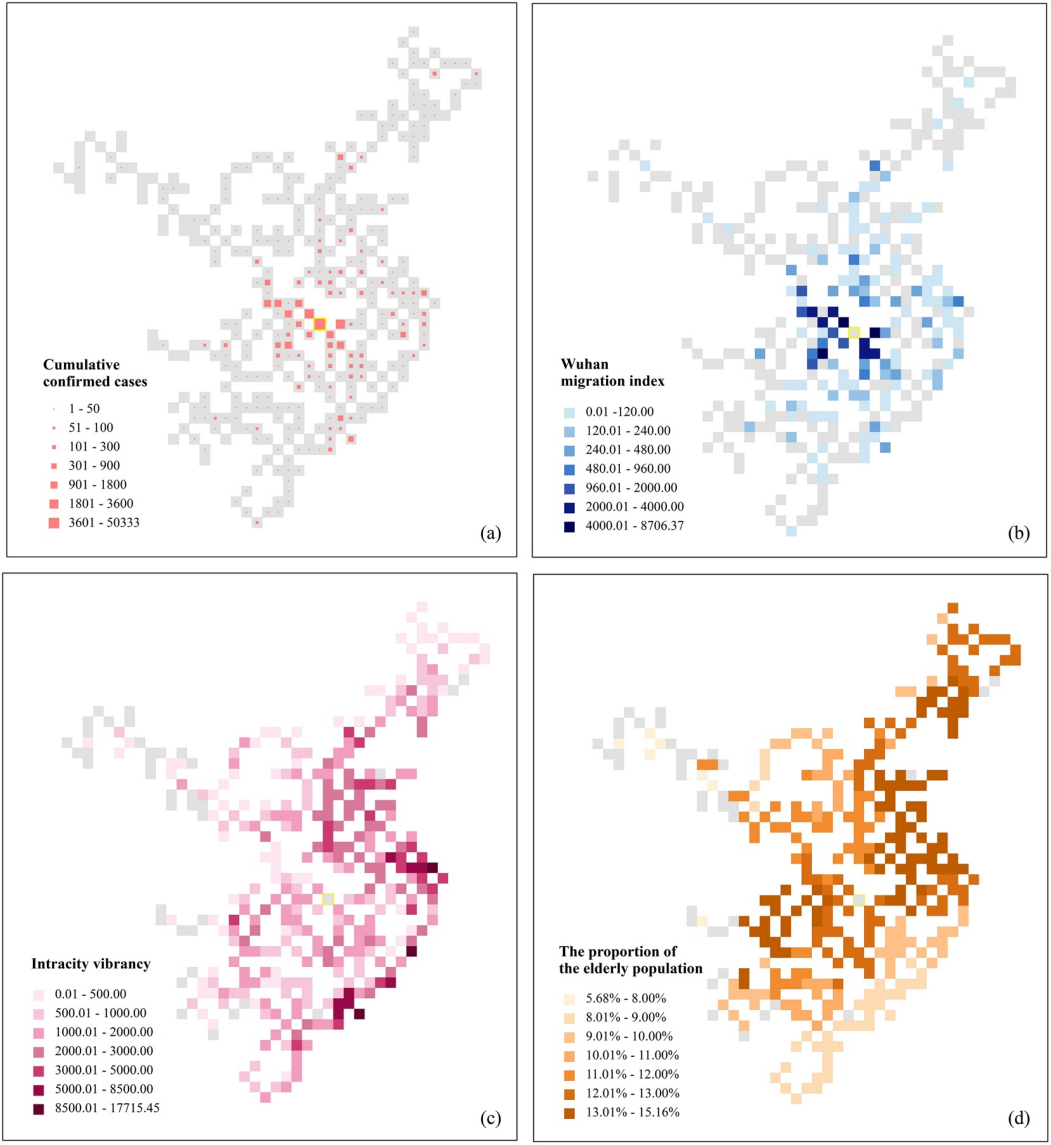
The point grid map for the variables: (a) Cumulative confirmed cases; (b) Wuhan migration index; (c) Intracity vibrancy; (d) The proportion of the elderly population.

This article also used a point grid map to visualize the cumulative confirmed cases (until April 30) and their potential influential factors (i.e., Wuhan migration index, intracity vibrancy, and the proportion of the elderly population), as shown in Figure 5. Intuitively, the spatial distribution of Wuhan migration index is consistent with the spatial distribution of the cumulative confirmed cases. The spatial distributions of intracity vibrancy and the proportion of the elderly population at the city level have exhibited their characteristics. The spatial‐temporal visualizations only show the macroscopic and qualitative relationships between the cumulative confirmed cases and the influential factors. The specific quantitative relationships between the cumulative confirmed cases of COVID‐19 and the influential factors are mainly dependent on the application of the mixed GWR method and the XGBoost regression model.

## 4 Factors of Influencing the COVID‐19 Epidemic

### 4.1 The Results of Mixed GWR

**Figure 6:**
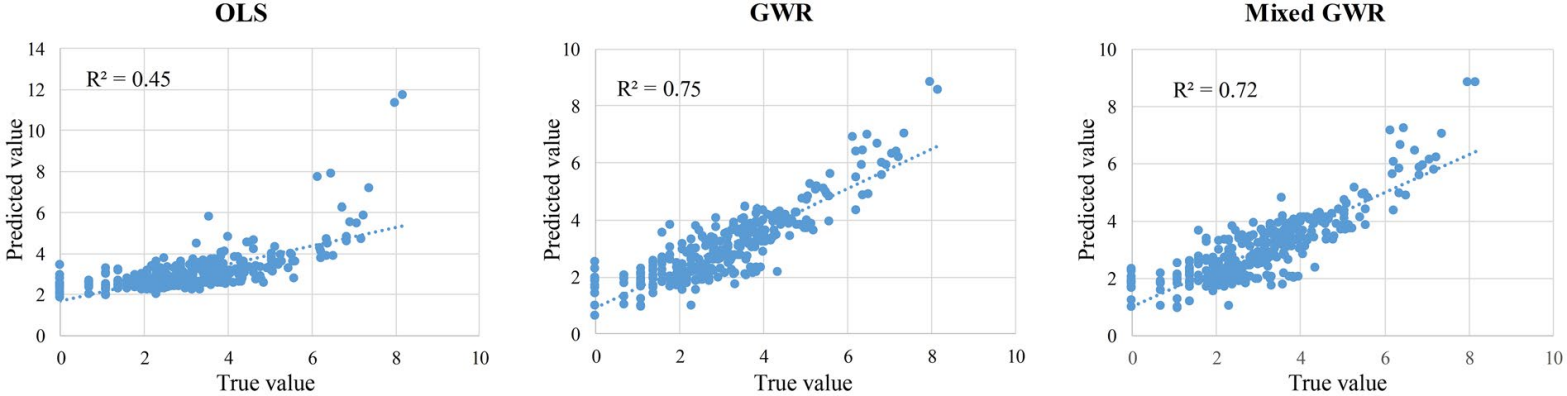
True vs. predicted values of ordinary least squares, geographically weighted regression (GWR), and mixed GWR.

This section explores the quantitative relationships between the cumulative confirmed cases of COVID‐19 and influential factors. First, we adopted Monte Carlo method (Number = 1,000) to test the statistical significance of spatial heterogeneity for each influential variable. The *p*‐values of intercept, Wuhan migration index, intracity vibrancy, and the proportion of the elderly population are 0.00, 0.00, 0.81, and 0.82, respectively, which illustrate that the estimated coefficients of intercept and Wuhan migration index have spatial heterogeneity with statistical significance and that the variables of intracity vibrancy and the proportion of the elderly population show global effects on COVID‐19. Thus, for the mixed GWR, the variables of intercept and Wuhan migration index are set as local variables, and intracity vibrancy and the proportion of the elderly population are set as global variables (i.e., spatial bandwidth = 316). Then, OLS, GWR, and mixed GWR models are implemented to model the relationships between the cumulative number of confirmed COVID‐19 cases and the influential factors. Table 1 lists the diagnostic information, and Figure 6 shows the goodness of fit between the true and predicted values of the three models. Although the GWR model gains the best results with the largest value of *R* ^2^ and the smallest value of residual sum of squares (RSS), the GWR result may exhibit a risk of overfitting to exaggerate the spatial heterogeneity. Moreover, the residuals of OLS and GWR are spatially autocorrelated, which shows a cluster pattern. Therefore, the mixed GWR is most applicable to model the relationships between the selected influential factors and the cumulative number of confirmed cases for the following reasons: (a) considering the statistical significance of spatial heterogeneity; (b) gaining a random pattern of residuals; and (c) obtaining the lowest value of AICc.

**Table 1:** Diagnostic Information of OLS, GWR, and Mixed GWR

Model	*R* ^2^	$R_{\text{adj}}^{2}$	RSS	AICc	Moran's *I* of residuals (*I*/*z*/*p*)
OLS	0.45	0.44	421.54	998.03	0.08/8.86/0.00^a^ (Cluster)
GWR	0.75	0.69	190.71	850.68	−0.02/−1.50/0.04^b^ (Cluster)
Mixed GWR	0.72	0.69	211.91	837.04	−0.01/−0.94/0.17 (Random)

Abbreviations: AICc, Akaike information criterion; GWR, geographically weighted regression; OLS, ordinary least squares; RSS, residual sum of squares.

^a^ and ^b^ Denote statistical significance at the 1% and 5% level, respectively.

Table 2 lists the estimated coefficients of the three models. OLS can reveal the global relationships between the cumulative confirmed COVID‐19 cases and the influential factors. All the estimated coefficients of the variables show that their impact on the cumulative confirmed cases significantly positive, suggesting that the overall number of cumulative confirmed cases gradually increases as the three variables increase. This finding is consistent with the expected results. Comparing the coefficients of GWR and mixed GWR, the intercept and Wuhan migration index show similar varying ranges. The spatial heterogeneity levels of intracity vibrancy and the proportion of the elderly population of GWR are greater than those of mixed GWR. According to Monte Carlo test, the results of GWR that calibrated using a single bandwidth (i.e., 48) may exaggerate the spatial heterogeneity level of relationships between COVID‐19 and independent variables, especially the variable of intracity vibrancy and the proportion of the elderly population. This article analyzes the spatially varying coefficients of variables based on the results of mixed GWR.

**Table 2:** The Estimated Coefficients of OLS, GWR, and Mixed GWR

Variables	OLS	GWR (bandwidth = 49)				Mixed GWR				
	Estimate	Mean	Min	Median	Max	Bandwidth	Mean	Min	Median	Max
Intercept	3.10^a^	4.19	3.18	4.07	7.42	48 (local)	4.11	3.14	3.98	6.96
Wuhan migration index	0.84^a^	4.88	0.32	3.79	22.12	48 (local)	5.32	0.35	4.43	19.23
Intracity vibrancy	0.54^a^	0.48	−1.17	0.44	1.87	316 (global)	0.22	0.22	0.22	0.22
The proportion of the elderly population	0.23^a^	0.09	−0.66	0.04	2.52	316 (global)	0.19	0.19	0.19	0.19

Abbreviations: GWR, geographically weighted regression; OLS, ordinary least squares.

^a^ Denotes statistical significance at the 1% level.

**Figure 7:**
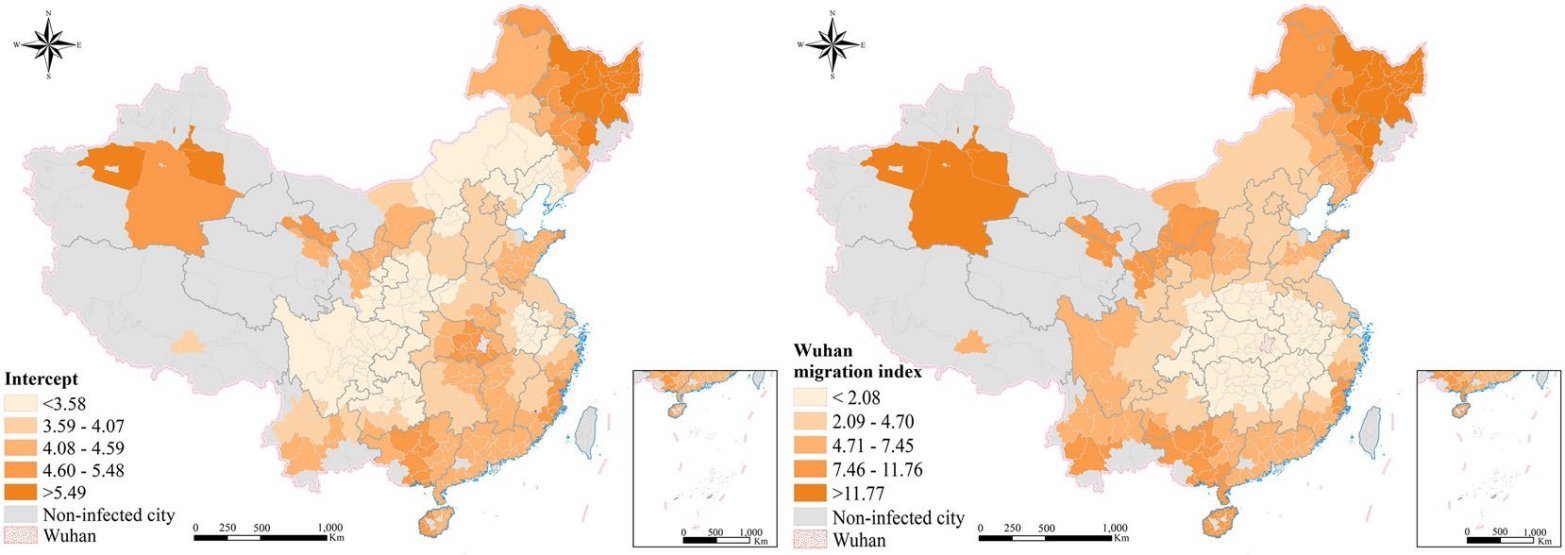
The local coefficients of intercept and Wuhan migration index.

For the mixed GWR, the variables of intracity vibrancy and the proportion of the elderly population are global, and the estimated coefficients are 0.22 and 0.19, respectively. Specifically, intracity vibrancy can reflect the degree of population exposure and intracity interaction. A higher intracity vibrancy value indicates a higher probability of people's contact with each other, thus increasing the risk of COVID‐19 infection. Research has shown that elderly people, especially those over 65 yr of age with more serious illnesses and poor self‐resistance, are susceptible to COVID‐19 (J. Wang et al., 2020). Therefore, the greater the demographic aging of a city is, the greater the risk and number of COVID‐19 infections will be. Figure 7 shows the local estimated coefficients of intercept and Wuhan migration index. For the variable of Wuhan migration index, it is apparent that the local estimated coefficients were all positive. These data indicate that the greater the migration index from Wuhan to the local city, the greater the number of people infected with COVID‐19. It is obvious that the distribution of coefficients is centered on Wuhan and increases to the periphery, and the degree of influence on the cumulative number of confirmed cases is significantly higher than that of other variables. The closer the city is to Wuhan, the population mobility from Wuhan has a relatively small impact on the number of confirmed COVID‐19 cases. Conversely, the farther the city is from Wuhan, the greater the influence. The relationship between cities is inseparable from population mobility but is not limited to it (F. Wang et., 2019). Therefore, we can reasonably hypothesize that cities closer to Wuhan do not exclude other channels of COVID‐19 infection, except for migrating populations. The above hypothesis can provide some enlightenment for the control and inspection of the flow of fresh products and other relief materials. Considering other factors that may have the effect of distance attenuation, the number of early diagnoses of COVID‐19 in cities far away from Wuhan was mainly influenced by the population migrating from Wuhan.

### 4.2 The Results of XGBoost

**Figure 8:**
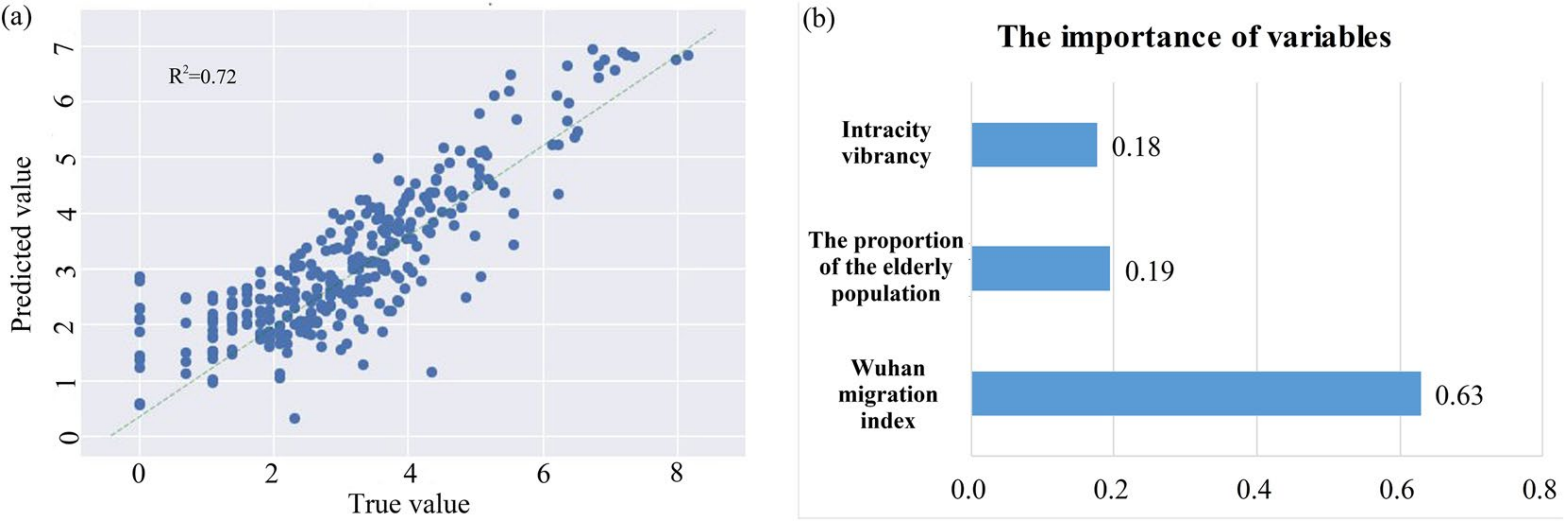
The results of extreme gradient boosting: (a) Predicted values and true values; and (b) The importance of the influential factors.

The optimal performance of the XGBoost model depends on parameter tuning (Chen & Guestrin, 2016). Figure 8 shows the results of XGBoost, and the *R* ^2^ is 0.72 and is similar to the results of mixed GWR. These results demonstrate that XGBoost can effectively complement the importance of influential factors that mixed GWR cannot reveal. The relative importance of the three variables as predictors for the cumulative confirmed cases of COVID‐19 is illustrated in Figure 8b. As expected, Wuhan migration index accounts for 63% of the overall importance of variables and is the most important explanatory variable in the XGBoost model. The significant correlation between the cumulative confirmed cases of COVID‐19 and human mobility has also been identified by some previous studies (Dey et al., 2020; Tian et al., 2020). The predictor of the proportion of the elderly population shows the second largest contribution to the cumulative confirmed cases of COVID‐19. The variable with the smallest contribution is intracity vibrancy (accounting for 18% of the overall importance). Therefore, the order of importance of the three influential factors on COVID‐19 is as follows: Wuhan migration index > the proportion of the elderly population > intracity vibrancy. The outbreak and spread of COVID‐19 is extremely rapid, especially in the early stages of the epidemic with the outflow of people from Wuhan playing a decisive role in the spread of COVID‐19. According to the order of importance of the three selected factors, our study also confirmed the importance and response emergency effects of the lockdown of Wuhan and can provide effective suggestions for the future prevention and control of related diseases.

## 5 Discussion and Conclusion

This article utilized an integrated approach of visualization, spatial regression, and machine learning to study COVID‐19 from a geographical perspective at the city level in China using multisource data sets. Specifically, the point grid map embedded in the calendar view is applied to visualize the spatial‐temporal distribution of confirmed COVID‐19 cases in China. Then, to reveal the factors that influence the spatial distribution of COVID‐19, the mixed GWR is used to explore the global or local effects of the factors, and the XGBoost regression model is adopted to identify the importance and contributions of the factors. The proposed framework is used to explore spatial‐temporal patterns and outlier patterns of COVID‐19 at the city level, which helps to improve our understanding of the evolution of COVID‐19 and provides useful information for supervising, preventing, and controlling the spread of COVID‐19. Moreover, the proposed framework integrating visualization, spatial regression, and machine learning can be applicable and extended to the study of spatial epidemiology in other regions at home and abroad.

In summary, the visualization and exploratory results of this study provide insights to control COVID‐19. There are three important findings based on the results of this article. First, cities in Hubei Province, especially Wuhan, exhibit a serious COVID‐19 situation at the beginning of 2020. In Wuhan, daily confirmed cases were concentrated from late January to mid‐March. However, in other cities of Hubei Province, daily confirmed cases were mainly concentrated from late January to February. Outside Hubei Province, the temporal patterns of COVID‐19 in Beijing, Shanghai, Guangzhou, Harbin, Shenzhen, Foshan, Chengdu, and Tianjin are different from those in other cities. For cities with unusual spatiotemporal patterns, the confirmed cases are generally all imported cases and import associated cases in March and April. For example, in Harbin, no new confirmed case was reported from late February to early April; however, since mid‐April, several confirmed cases have been reported. Second, the confirmed COVID‐19 cases at the city level in China are significantly influenced by mobility, urban vibrancy, and the proportion of the aging population. From the perspective of statistical significance, the human migration before Wuhan closure has the greatest heterogeneity and presents the effect of distance decay. The overall stability of the impact of urban vitality after Wuhan closure and the aging population indicate that the outbreak was prevented by the concerted efforts of the whole China without significant spatial differences and variations. A resurgence of the epidemic occurred in some cities of China just before the Spring Festival in 2020, and the results of the heterogeneity test and mixed GWR have confirmed the importance of population migration at the city level in addition to physical protection at the individual level. Considering the spatial variations of influential factors is important to effectively deal with the uncertainty and risk of emergencies, which is important for formulating emergency policies. Finally, the order of importance of the three influential factors is as follows: Wuhan migration index > the proportion of the elderly population > intracity vibrancy. In sum, one of the most effective ways to control and defeat COVID‐19 involve controlling the source of infection, reducing crowd gatherings, cutting off the transmission route, and protecting vulnerable groups.

Although this article has new findings and strengths, there are still limitations worth mentioning. This article only visualized the spatial‐temporal pattern of confirmed COVID‐19 cases. In addition, most cities have begun to resume work and production, and information about the population mobility between and within cities is important for emergency decision‐making and reasonable allocation of medical resources (Zhou et al., 2020). Therefore, the spatial and temporal patterns of population mobility will be visualized and analyzed in the future. The influential factors selected in this article are limited to three human‐related variables. All the conclusions are based on the selected variables. However, changes in temperature, humidity, and air quality may affect the spread of COVID‐19 (Ma et al., 2020; Ogen, 2020). Influential factors may also change at different stages of COVID‐19. For instance, international air connectivity and whether a city is a border city may represent important factors in the current stage. New potential influential factors should be added in future analyses. In addition, the data of the elderly population in this article are available at the province level, which may cause some loss of model accuracy. We will identify other proxies to measure the susceptible population. Finally, this article mainly studied the cumulative number of confirmed cases, but COVID‐19 infection has an incubation period wherein the individual is contagious during this period. Meanwhile, the population of each city is unevenly distributed. Therefore, it is necessary to include suspected cases and use the infection rate indicator for analysis in subsequent studies.

## Conflict of Interest

The authors declare no conflicts of interest relevant to this study.

## Supporting information

Figure S1

## Data Availability

The COVID‐19 case data are publicly available at https://doi.org/10.7910/DVN/MR5IJN. The mobility data from Wuhan are publicly available at https://doi.org/10.7910/DVN/FAEZIO. The population data of 2020 are publicly available from the Socioeconomic Data and Applications Center (https://sedac.ciesin.columbia.edu).
