# Supplementary material for: Analyzing COVID‐19 Using Multisource Data: An Integrated Approach of Visualization, Spatial Regression, and Machine Learning
Source: Geohealth. 2021 Aug 1;5(8):e2021GH000439. doi: 10.1029/2021GH000439 (PMC8335962; doi:10.1029/2021GH000439)
Supplement: Supplementary file 1 — Figure S1 [file GH2-5-e2021GH000439-s001.pdf]

GeoHealth

Supporting Information for

Analyzing COVID-19 Using Multisource Data: An Integrated Approach of Visualization, Spatial Regression and Machine Learning

Chao Wu[1,2], Mengjie Zhou[3,4], Pengyu Liu[1], and Mengjie Yang[3]

[1] School of Geographic and Biologic Information, Nanjing University of Posts and Telecommunications, Nanjing 210023, China, [2] Smart Health Big Data Analysis and Location Services Engineering Lab of Jiangsu Province, Nanjing University of Posts and Telecommunications, Nanjing 210023, China, [3] College of Resources and Environmental Science, Hunan Normal University, Changsha 410081, China, [4] Key Laboratory of Geospatial Big Data Mining and Application, Hunan Province, Changsha 410000, China.

Contents of this file

Figure S1

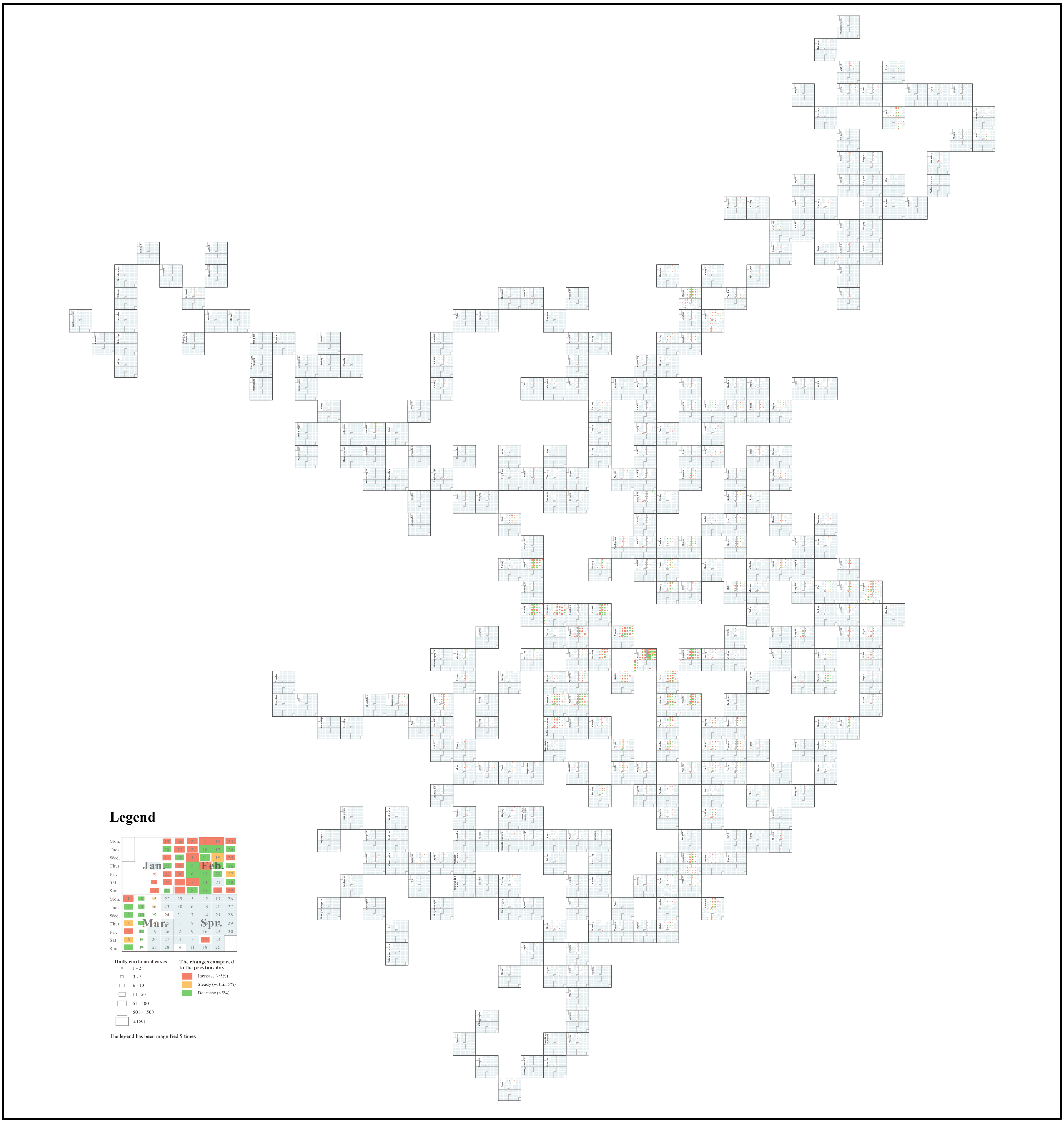

Figure S1. The spatial-temporal visualization of daily confirmed cases of COVID-19.
